# Augmented Atherogenesis in LDL Receptor Deficient Mice Lacking Both Macrophage ABCA1 and ApoE

**DOI:** 10.1371/journal.pone.0026095

**Published:** 2011-10-11

**Authors:** Bart Lammers, Ying Zhao, Menno Hoekstra, Reeni B. Hildebrand, Dan Ye, Illiana Meurs, Theo J. C. Van Berkel, Miranda Van Eck

**Affiliations:** Division of Biopharmaceutics, Leiden/Amsterdam Center for Drug Research, Gorlaeus Laboratories, Leiden University, Leiden, The Netherlands; King's College London, University of London, United Kingdom

## Abstract

**Aim:**

ABCA1 protects against atherosclerosis by facilitating cholesterol efflux from macrophage foam cells in the arterial wall to extracellular apolipoprotein (apo) A-I. In contrast to apoA-I, apoE is secreted by macrophages and can, like apoA-I, induce ABCA1-mediated cholesterol efflux. Yet, the combined effect of macrophage ABCA1 and apoE on lesion development is unexplored.

**Methods and Results:**

LDL receptor knockout (KO) mice were transplanted with bone marrow from ABCA1/apoE double KO (dKO) mice, their respective single KO's, and wild-type (WT) controls and were challenged with a high-fat/high-cholesterol diet for 9 weeks. In vitro cholesterol efflux experiments showed no differences between ABCA1 KO and dKO macrophages. The serum non-HDL/HDL ratio in dKO transplanted mice was 1.7-fold and 2.4-fold (p<0.01) increased compared to WT and ABCA1 KO transplanted mice, respectively. The atherosclerotic lesion area in dKO transplanted animals (650±94×10^3^ µm^2^), however, was 1.9-fold (p<0.01) and 1.6-fold (p<0.01) increased compared to single knockouts (ABCA1 KO: 341±20×10^3^ µm^2^; apoE KO: 402±78×10^3^ µm^2^, respectively) and 3.1-fold increased (p<0.001) compared to WT (211±20×10^3^ µm^2^). When normalized for serum cholesterol exposure, macrophage ABCA1 and apoE independently protected against atherosclerotic lesion development (p<0.001). Moreover, hepatic expression levels of TNFα and IL-6 were highly induced in dKO transplanted animals (3.0-fold; p<0.05, and 4.3-fold; p<0.001, respectively). In agreement, serum IL-6 levels were also enhanced in ABCA1 KO transplanted mice (p<0.05) and even further enhanced in dKO transplanted animals (3.1-fold as compared to ABCA1 KO transplanted animals; p<0.05).

**Conclusions:**

Combined deletion of macrophage ABCA1 and apoE results in a defect in cholesterol efflux and, compared to ABCA1 KO transplanted mice, elevated serum total cholesterol levels. Importantly, these mice also suffer from enhanced systemic and hepatic inflammation, together resulting in the observed augmented atherosclerotic lesion development.

## Introduction

Accumulation of cholesterol in macrophages leads to the formation of foam cells, a crucial event in the development of atherosclerotic lesions. Because macrophages are incapable of limiting the uptake of lipoproteins, these cells rely on reverse cholesterol transport (RCT) for maintaining cellular cholesterol homeostasis [1], [2]. We have shown that apolipoprotein (apo) E as well as the ATP-binding cassette (ABC) transporters A1 and G1 are key players in the efflux of cholesterol from macrophages, the first step in RCT and protection against atherosclerosis [3], [4], [5]. ABCA1 facilitates cholesterol efflux to lipid-poor apolipoproteins [6] like apoA-I, resulting in the formation of lipidated HDL. Subsequently, ABCG1 mediates the efflux of cellular cholesterol to these mature HDL particles [7], [8]. In addition, ABCA1 can modulate the secretion of apoE [9]. Secreted apoE by macrophages facilitates cellular cholesterol efflux [10] both in the presence and absence of extracellular cholesterol acceptors [11]. Although both pro- and anti-atherogenic functions for macrophage apoE have been described [12], [13], [14], [15], we recently showed that macrophage apoE protects against atherosclerotic lesion development, independent of ABCG1 [5] in LDL receptor knockout (LDLr KO) mice. Moreover, very recently, Zanotti et al. showed that expression of apoE only in macrophages is sufficient to promote RCT, emphasizing the pivotal anti-atherogenic role for macrophage apoE [16]. Importantly, macrophage apoE-mediated cholesterol efflux was shown to be independent of ABCA1 in apoE expressing J774 macrophages [17], suggesting that the atheroprotective effects of macrophage ABCA1 are independent of apoE production by macrophages. However, in primary human monocyte-derived macrophages and THP-1 cells it was shown that apoE secretion from macrophages is promoted by ABCA1 [9].

To investigate the possible interaction between ABCA1 and apoE in promoting macrophage cholesterol efflux and their combined roles in atherogenesis, we generated mice deficient for both ABCA1 and apoE and performed a bone marrow transplantation (BMT) experiment in LDLr KO mice. Our results evidently show that transplantation of LDLr KO mice with either ABCA1 KO or apoE KO bone marrow resulted in a moderate increase in atherosclerotic lesion development, while combined deletion of ABCA1 and apoE in bone marrow-derived cells led to a more dramatic increase in atherosclerosis.

## Methods

### Animals and Bone Marrow Transplantation

**Figure 1:**
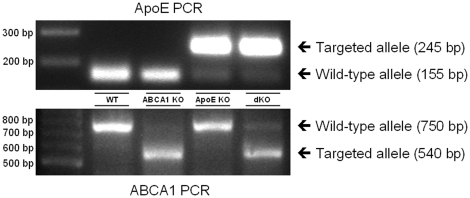
Successful disruption of macrophage ABCA1 and apoE by bone marrow transplantation. RT-PCR on genomic DNA of bone marrow isolated from ABCA1 and/or apoE KO transplanted LDLr KO mice and their WT transplanted controls at 17 weeks after transplantation.

ABCA1 KO [18] and apoE KO (The Jackson Laboratory, Bar Harbor, ME) mice (both more than 7 times backcrossed onto a C57BL/6J background) were mated to generate F1 heterozygotes. Heterozygote F1 animals were crossbred to obtain ABCA1^−/−^/apoE^+/+^ (ABCA1 KO), ABCA1^+/+^/apoE^−/−^ (apoE KO), ABCA1^−/−^/apoE^−/−^ (dKO), and ABCA1^+/+^/apoE^+/+^ (WT) mice, which were used as donors for the bone marrow transplantation. These donor mice were anaesthetized subcutaneously with a mix of 70 mg/kg body weight xylazine, 1.8 mg/kg bodyweight atropine and 350 mg/kg body weight ketamine. Animals were subsequently sacrificed by cervical dislocation. Bone marrow cells were then isolated from the femurs and tibias from these mice. Homozygous C57BL/6J LDL receptor knockout (LDLr KO) mice were obtained from The Jackson Laboratory as mating pairs and bred at the Gorlaeus Laboratory, Leiden, The Netherlands. Bone marrow transplantations to male LDLr KO mice were performed as described [19]. Briefly, irradiated recipients (≥11 per group) received 5×10^6^ bone marrow cells by intravenous injection into the tail vein. After a recovery period of 8 weeks, the animals were challenged with a high-fat/high-cholesterol (HF/HC; 1.25% cholesterol, 21% fat (Abdiets, Woerden, the Netherlands)) diet for 9 weeks to induce atherosclerotic lesion development. At 17 weeks after transplantation, mice were anaesthetized subcutaneously with a mix of 70 mg/kg body weight xylazine, 1.8 mg/kg bodyweight atropine and 350 mg/kg body weight ketamine. Animals were subsequently sacrificed by cervical dislocation. The hematologic chimerism of the transplanted LDLr KO mice was confirmed in genomic DNA from bone marrow by PCR analysis (Fig. 1). Animal experiments were approved by the Ethics Committee for Animal Experiments of Leiden University (permit number 08015) and performed at the Gorlaeus Laboratories of the Leiden/Amsterdam Center for Drug Research in accordance with the National Laws and the Directive 2010/63/EU of the European Parliament.

### Histological Analysis of the Aortic Root, Aortic Arch, and Spleen

To analyze the development of atherosclerosis at the aortic root and aortic arch, transplanted LDLr KO mice were euthanized after 9 weeks of feeding the HF/HC diet. The arterial tree was perfused in situ with PBS (100 mm Hg) for 10 min via a cannula in the left ventricular apex. The heart plus aortic root and the aortic arch were excised and stored in 3.7% neutral-buffered formalin (Formal-fixx; Shandon Scientific Ltd, Runcorn, UK). Serial sections (10 µm) of the aortic root were cut using a Leica CM3050S cryostat. The atherosclerotic lesion areas in oil red-O stained cryostat sections of the aortic root and whole aortic arches were quantified using the Leica image analysis system, consisting of a Leica DMRE microscope coupled to a video camera and Leica Qwin Imaging software (Leica Ltd, Cambridge, UK). Mean lesion area (in µm^2^) was calculated from 10 consecutive oil red-O stained sections of the aortic root, starting at the appearance of the tricuspid valves. Sections were stained immunohistochemically for the presence of macrophages using a rat MOMA-2 antibody, dilution 1∶50 (Serotec Ltd, Oxford, UK). Goat anti-rat coupled to horse radish peroxidase (HRP) (1∶100) (Dako, Glostrup, Denmark) was used as a secondary antibody and Nova red substrate (Vector Laboratories, Burlingame, California) was used for visualization of HRP. Collagen content of the plaques was determined after Masson's Trichrome staining (Sigma Diagnostics, St Louis, MO, USA). Relative plaque area in the aortic arch was quantified *en face* calculating the ratio of atherosclerotic lesions to the surface of the entire aortic arch. In addition, 7 µm cryosections of formalin-fixed spleen from the transplanted LDLr KO mice were prepared and stained for lipid accumulation using oil red-O and counterstained with hematoxylin.

### Lipid Analyses

Eight weeks after bone marrow transplantation, 100 µL of blood was drawn from each individual mouse by tail bleeding after an overnight fasting period. Upon sacrifice, 17 weeks after bone marrow transplantation, blood was collected by retro-orbital venous plexus puncture after an overnight fasting period. Lipid analyses were performed as described [19].

Lipoprotein profiles from all mice were determined by fractionation of 50 µL samples of serum, pooled from 2 mice per genotype, using a Superose 6 column (3.2×300 mm, Smart-System; Pharmacia, Uppsala, Sweden). Total cholesterol (TC) content of the effluent was determined as described [19], and calculated as a percentage of serum TC. Splenic free cholesterol (FC), cholesteryl ester (CE) and triglyceride (TG) content were determined as described [19] after extraction according to Bligh and Dyer [20] and dissolving the lipids in 2% Triton X-100. ApoE Western blot analysis of pooled VLDL and LDL fractions was performed by running equal amounts of total cholesterol per lipoprotein (VLDL: 3.8 mg, LDL: 4.6 mg) on a 15% SDS-PAGE gel. ApoE was detected using a rabbit-anti-mouse apoE polyclonal antibody (SB Rabbit 67-AH). As a secondary antibody a goat-anti-rabbit IgG-HRP (Jackson ImmunoResearch, Suffolk, UK) was used.

### Cellular Cholesterol Efflux

For isolation of bone marrow-derived macrophages (BMDM), femurs and tibias were lavaged with PBS. Bone marrow cells were plated in DMEM/20% FCS/1% penicillin/1% streptomycin and differentiated into macrophages by addition of 30% L929 cell-conditioned media (as a source of M-CSF) for 7 days. Subsequently, BMDM were incubated with 0.5 µCi/mL ^3^H-cholesterol in DMEM/0.2% fatty acid free BSA and 30 µg/mL non-labeled cholesterol for 24 h at 37°C. Cholesterol efflux was determined after incubating the cells in DMEM/0.2% fatty acid free BSA in the absence or presence of 10 µg/mL apoAI (Calbiochem, La Jolla, CA, USA) or 50 µg/mL human HDL, isolated according to Redgrave *et al.* [21]. Radioactivity in the medium and the cells was determined by scintillation counting after 24 h of incubation. Cholesterol efflux is expressed as the percentage of total cell ^3^H-cholesterol present in the medium after 24 h. Basal efflux to BSA (in the absence of ApoA-I and HDL) has been subtracted from the data shown.

### mRNA Expression Analysis by Real Time PCR

Total RNA from BMDM was isolated using the guanidinium thiocyanate (GTC) method [22] and reverse transcribed using a RevertAid M-MuLV enzyme (Fermentas, Burlington, Canada). Relative mRNA expression was measured from the following genes: scavenger receptor class B type I (SR-BI), LDL receptor-related protein 1 (LRP1), 3-hydroxy-3-methyl-glutaryl-CoA reductase (HMG-CoA reductase), microsomal triglyceride transfer protein (MTP), ATP-binding cassette transporter sub-family G member 1 (ABCG1), apolipoprotein (apo) B and E, lipoprotein lipase (LPL), fatty acid synthase (FAS), interleukin 6 (IL-6), tumor necrosis factor α (TNFα), and diacylglycerol acyltransferase-1 (DGAT-1). The mRNA expression levels were assessed by real time PCR (ABI PRISM 7500; Applied Biosystems, Foster City, CA) using SYBR Green technology (Applied Biosystems). Housekeeping genes RPS13, 36B4, and HPRT were used as a control. Primer sequences are available upon request.

### Determination of Serum Cytokine Concentrations

Murine IL-6 and IFNγ serum levels were assayed using an instant ELISA kit (eBioscience, Hatfield, UK) according to the manufacturer's protocol. Murine IL-10 and TNFα serum levels were assayed using an instant ELISA kit (BD Biosciences, Erembodegem, Belgium) according to the manufacturer's protocol.

### Statistical Analysis

Statistically significant differences among the means of the different populations were tested using analysis of variance (ANOVA) and when specifically indicated the unpaired Student's t-test (GraphPad InStat and Prism software). The Student-Newman-Keuls multiple comparison test was performed after ANOVA. Two-way ANOVA was used to check possible interactions. The probability level (alpha) for statistical significance was set at 0.05. Results are expressed as an average ± SEM.

## Results

### Shift Toward a More Pro-Atherogenic Lipoprotein Profile in ABCA1/apoE dKO Transplanted LDLr KO Mice

Cholesterol levels were measured at 8 weeks (chow diet) and at 17 weeks (HF/HC diet, containing 1.25% cholesterol and 21% fat) after BMT (Table 1). On chow diet, free cholesterol (FC) levels were moderately increased in apoE KO transplanted mice (15%, p<0.01). However, no effect of ABCA1 and/or apoE deletion in bone marrow-derived cells was observed on serum total cholesterol (TC) levels. After 9 weeks of feeding the HF/HC diet, serum cholesterol levels of the control mice and the apoE KO transplanted mice increased ≈3-fold. However, in agreement with previous studies [3], [23], deletion of ABCA1 in bone marrow cells resulted in an attenuated 1.7-fold increase in plasma TC levels upon feeding an atherogenic diet compared to WT controls (p<0.001). Importantly, although ABCA1/apoE dKO transplanted mice still exhibited an attenuated rise in TC levels compared to WT controls (2.4-fold; p<0.01), these levels were significantly higher compared to levels of ABCA1 KO transplanted mice (1.4-fold; p<0.05).

**Table 1:** Serum lipid and lipoprotein levels were measured at 8 weeks (chow diet) and 17 weeks (HF/HC diet) after transplantation.

Donor BM	Time (wks)	Diet	FC (mg/dL)	TC (mg/dL)	Non-HDL-C (mg/dL)	HDL-C (mg/dL)	Non-HDL/HDL ratio
WT	8	Chow	81±2	317±15	n.d	n.d	n.d
	17	HF/HC	267±14	1021±75	929±89	92±11	10±1
ABCA1 KO	8	Chow	75±5	294±15	n.d	n.d	n.d
	17	HF/HC	148±13***	493±41***	430±56***	63±5**	7±1
apoE KO	8	Chow	93±3**	323±14	n.d	n.d	n.d
	17	HF/HC	303±17	1030±84	969±67	61±5*	17±3##
dKO	8	Chow	83±3	294±13	n.d.	n.d	n.d
	17	HF/HC	216±18* ##	691±66** #	647±106*	44±7***	17±4##

Data represent mean ± SEM of ≥10 mice.

* p<0.05,

** p<0.01,

*** p<0.001 compared to WT;

# p<0.05,

## p<0.01 compared to ABCA1 KO; n.d. = not determined.

**Figure 2:**
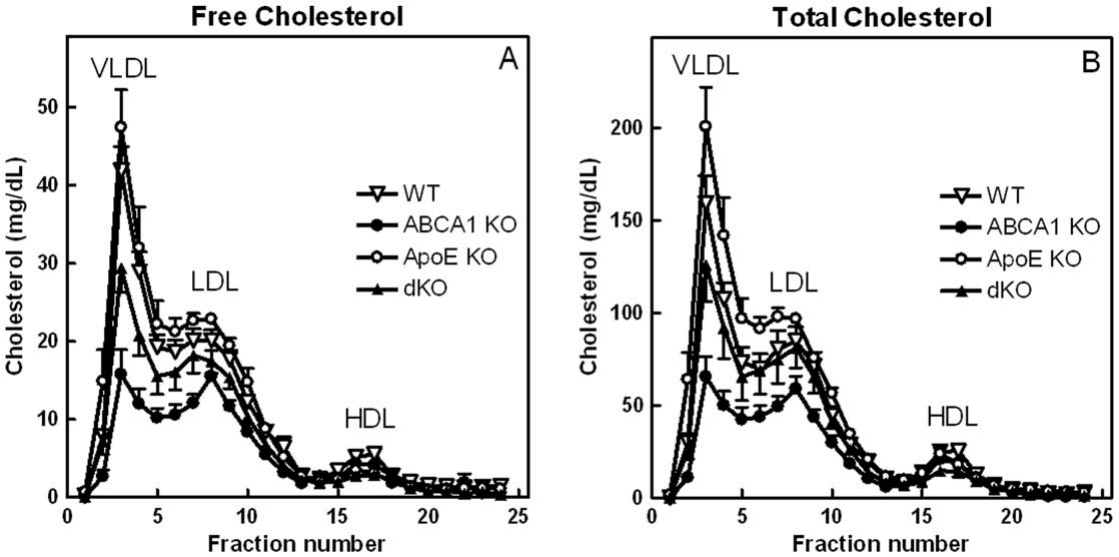
Lipoprotein profiles from ABCA1 and/or apoE KO transplanted LDLr KO mice and their WT transplanted controls. Fifty µL samples of serum, pooled from 2 mice per genotype, collected after 9 weeks of HF/HC feeding, were subjected to FPLC fractionation. Distribution of serum FC and TC over the fractions (25 µL) was determined enzymatically. Values represent the means of n≥5 samples per group. 1 sample corresponds to pooled serum of 2 mice.

The distribution of TC among serum lipoproteins was analyzed by liquid chromatography (Fig. 2). The observed decrease in cholesterol levels of ABCA1 KO transplanted mice upon feeding the HF/HC diet was mainly caused by a 54% decrease in cholesterol associated with VLDL and LDL (non-HDL-C; p<0.001; Table 1). However, HDL-C levels in these mice were only reduced by 32%, which resulted in a decreased non-HDL/HDL ratio (Table 1). In contrast, the non-HDL/HDL ratio of apoE KO transplanted mice was increased compared to WT controls (1.7-fold; Table 1), since HDL-C was reduced by 34% (p<0.05; Table 1), whereas non-HDL-C levels remained stably high. In agreement with ABCA1 KO transplanted mice, non-HDL-C of apoE/ABCA1 dKO transplanted animals was reduced compared to WT controls (30%; p<0.05; Table 1). However, also a dramatic 52% reduction was observed in HDL-C levels of these mice (p<0.001; Table 1), which resulted in an increased non-HDL/HDL ratio (2.4-fold; p<0.01 compared to ABCA1 KO; Table 1), suggesting a more pro-atherogenic index.

### No Differences in Serum VLDL/LDL ApoE Concentrations after HF/HC Diet Feeding

**Figure 3:**
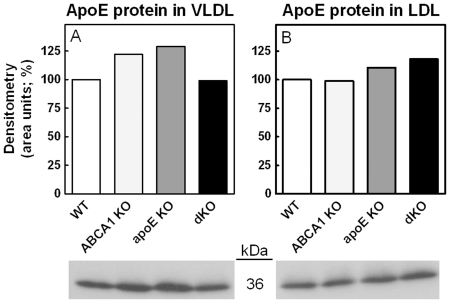
ApoE protein content in VLDL and LDL fractions from ABCA1 and/or apoE KO transplanted LDLr KO mice and their WT transplanted controls. Western blots showing apoE protein expression in VLDL (A) and LDL (B) isolated from ABCA1 KO and/or apoE KO transplanted LDLr KO animals and WT transplanted controls after feeding the HF/HC diet for 9 weeks. Samples were adjusted for cholesterol content. The density of the bands was determined using quantification software (Image J). n≥5 samples per group (pooled). WT values were set at 100%.

ApoE is essential for clearance of lipoproteins from the circulation via the LDLr or LDL-receptor-related protein (LRP1). To investigate if normalization of the HF/HC diet-induced increase in VLDL/LDL is the consequence of a lower apoE content on these particles, apoE was determined in the lipoprotein fractions by Western blotting and normalized for cholesterol in the fractions (Fig. 3)

No apparent differences were observed in the apoE content of VLDL and LDL between the different groups of mice, indicating that disruption of apoE production by bone marrow-derived cells does not impair the availability of apoE on the particles for clearance. No apoE was visible in the HDL fraction of the different groups.

### Increased Lipoprotein Lipase mRNA Expression in Liver

In order to further investigate the possible mechanisms regarding the observed differences in serum TC levels, we quantified mRNA expression levels of key liver enzymes in fatty acid and triglyceride synthesis (FAS and DGAT-1, respectively), cholesterol efflux (ABCG1) and synthesis (HMG-CoA reductase), apolipoproteins (apo) B and E, and genes involved in lipoprotein assembly (MTP) and uptake (SR-BI, LRP1), but found no differences. However, hepatic lipoprotein lipase (LPL) expression was 1.9-fold increased in ABCA1 KO transplanted animals as compared to dKO transplanted animals (p<0.05). LPL is essential for the lipolysis of lipoproteins. Induction of LPL leads to rapid clearance of VLDL and chylomicrons. Therefore, the observed attenuation of LPL expression in the dKO transplanted mice might have contributed to the increased TC levels as compared to ABCA1 KO transplanted animals.

### Enlarged, Cholesteryl Ester Enriched Spleens in ABCA1/apoE dKO Transplanted Mice

**Figure 4:**
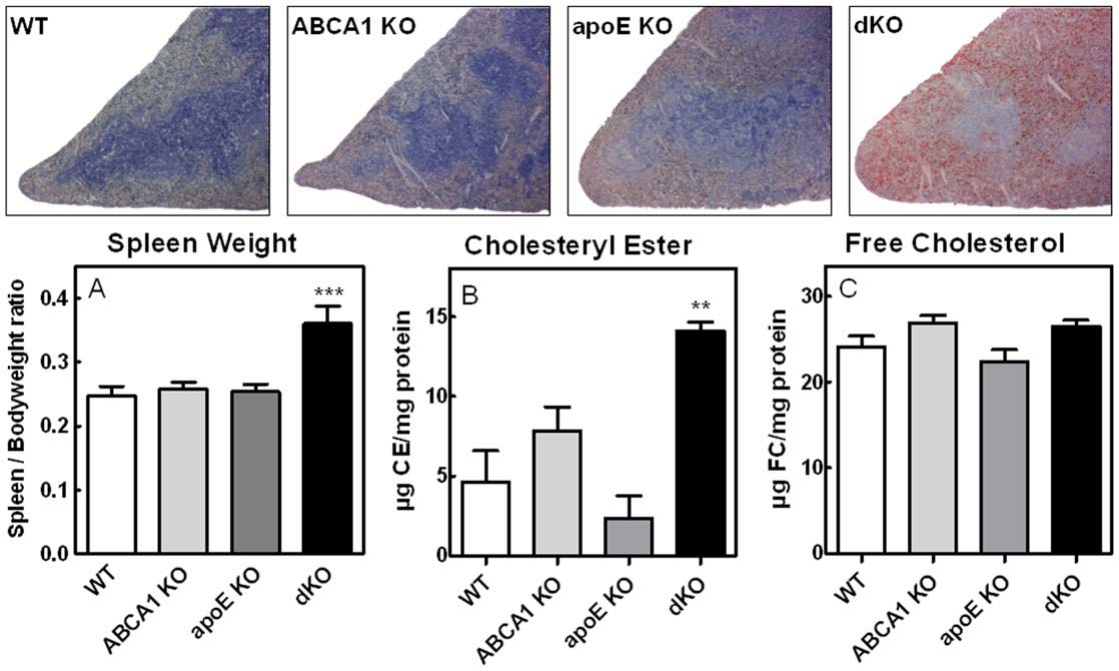
Increased CE content in enlarged spleens from ABCA1/apoE dKO transplanted LDLr KO mice. Representative cross sections of spleens from the bone marrow transplanted LDLr KO mice stained with oil red-O for the detection of neutral lipids and counterstained with hematoxylin (magnification 50×; upper panels). Spleen weight to bodyweight ratio is increased in ABCA1/apoE dKO transplanted LDLr KO mice (A). Values represent the means of n≥11 mice ± SEM per group. Quantification of cholesteryl ester (CE) and free cholesterol (FC) content of spleens (B and C, respectively). Values represent the means of n≥5 mice ± SEM per group. **p<0.01, ***p<0.001 as compared to WT transplanted controls.

Upon sacrifice (17 weeks after BMT; 9 weeks HF/HC diet feeding), spleens from all mice were examined. While both single KO transplanted groups showed no differences in spleen weight compared to WT controls, spleens from dKO transplanted animals were enlarged by 39% (p<0.001; Fig. 4). To further investigate this, spleen sections were stained with oil red-O to visualize lipid accumulation. No apparent staining was visible in spleens from WT controls and apoE KO transplanted mice, while spleens from ABCA1 KO transplanted mice stained mildly for lipids (Fig. 4). In contrast, mice transplanted with ABCA1/apoE dKO bone marrow exhibited a heavily lipidated spleen, predominantly in the red pulp, explaining the large increase in size. To further investigate the splenic lipid composition, we isolated the lipid content and measured FC, CE and TG levels. In agreement with the lipid accumulation visualized by oil red-O staining, spleens from dKO transplanted mice revealed a 3.0-fold (p<0.01; Figure 4B) increase in CE levels. FC and TG content of these spleens were not significantly different. No lipid accumulation was observed in other tissues.

### Increased Atherosclerosis in ABCA1/apoE dKO Transplanted LDLr KO Mice

**Figure 5:**
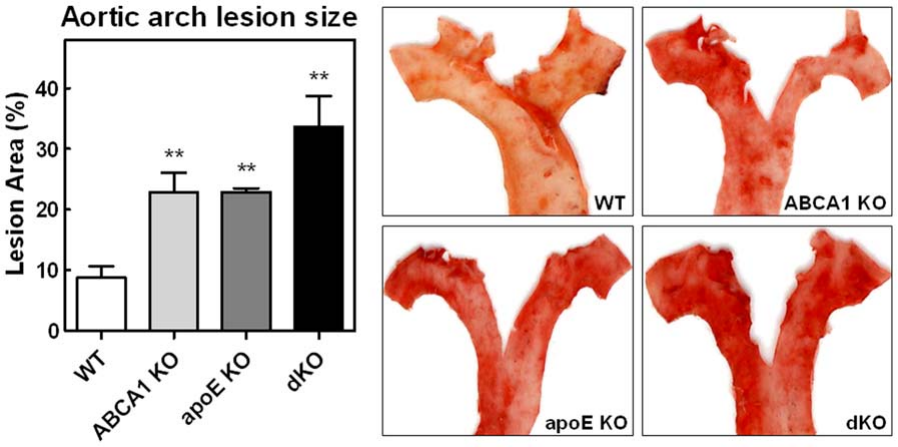
Analysis of atherosclerosis in the aortic arch of bone marrow transplanted LDLr KO mice after 9 weeks of HF/HC diet feeding. Relative plaque area in the aortic arch was quantified *en face* calculating the ratio of atherosclerotic lesions to the surface of the entire aortic arch (left panel). Representative pictures (right panels). **P<0.01 as compared to WT transplanted controls.

**Figure 6:**
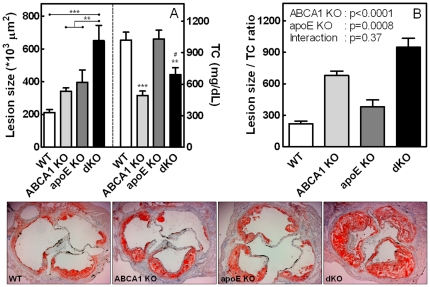
Analysis of atherosclerosis in the aortic root of bone marrow transplanted LDLr KO mice after 9 weeks of HF/HC diet feeding. Quantification of atherosclerotic lesion sizes in the aortic root and TC levels in bone marrow transplanted LDLr KO mice after 9 weeks of HF/HC diet feeding (A). Lesion size divided by TC levels show cholesterol independent effects on lesion development (B). Representative cross-sections, stained with oil red-O (magnification 50×; lower panels).Values are the means of 10 aortic root sections of individual mice (n≥11 mice ± SEM per group. **p<0.01, ***p<0.001 as compared to WT transplanted controls, ^#^p<0.05 as compared to ABCA1 KO transplanted animals.

After feeding the HF/HC diet for 9 weeks, lesion development in the aortic root and the aortic arch of the transplanted animals was analyzed. *En face* quantification of the aortic arch lesions revealed a 2.5-fold increased lesion area in single KO transplanted mice compared to WT controls (p<0.01; WT: 9±2%, ABCA1 KO: 23±3%, apoE KO: 22±1%) and a striking 3.8-fold increase in dKO transplanted animals (p<0.01; dKO: 34±5%; Fig. 5). In agreement, quantification of the lesion sizes in oil red-O stained sections of the aortic root showed that single deletion of macrophage ABCA1 or apoE led to a 1.6- or 1.9-fold increase in the mean atherosclerotic lesion size, respectively, compared to lesions in WT transplanted animals (341±20×10^3^ µm^2^ in mice reconstituted with ABCA1 KO bone marrow and 402±78×10^3^ µm^2^ in animals transplanted with apoE KO bone marrow, compared to 211±20×10^3^ µm^2^ in mice reconstituted with WT bone marrow; Fig. 6A). This however failed to reach statistical significance by ANOVA. As the current study consisted of four groups, ANOVA is the appropriate test to check for significant differences. Previous studies [3], [12], [13], however, used two groups (WT vs. KO), which only required a t-test. When the same t-test is used, both ABCA1 KO and apoE KO transplanted animals showed a significant increase in atherosclerotic lesion size compared to mice reconstituted with WT bone marrow (p<0.001 and p = 0.042, respectively). Combined deletion of both ABCA1 and apoE in bone marrow-derived cells in LDLr KO mice induced a significant 3.1-fold (p<0.001; ANOVA) increase in the mean atherosclerotic lesion size compared to WT transplanted animals (650±94×10^3^ µm^2^ compared to 211±20×10^3^ µm^2^). In addition, the lesion size in the dKO transplanted animals was also significantly larger compared to mice transplanted with single ABCA1 KO or apoE KO bone marrow (1.9- and 1.6-fold, respectively; p<0.01; ANOVA).

No differences in macrophage content in the aortic root plaques were observed (WT: 143±19×10^3^ µm^2^, ABCA1 KO: 136±19×10^3^ µm^2^, apoE KO: 198±27×10^3^ µm^2^, dKO: 139±21×10^3^ µm^2^). As expected, the collagen content was significantly increased in plaques from dKO transplanted mice, indicating more advanced lesions (p<0.01 to single KO transplanted mice and WT controls (WT: 3.3±1.2×10^3^ µm^2^, ABCA1 KO: 6.8±1.8×10^3^ µm^2^, apoE KO: 3.6±0.8×10^3^ µm^2^, dKO: 26±9.3×10^3^ µm^2^).

Interestingly, although single deletion of macrophage ABCA1 or apoE showed a comparable increase in atherosclerotic lesion development, TC levels between these groups were dramatically different (Fig. 6A). In humans, TC levels are correlated with atherosclerosis and are therefore a widely accepted risk factor for atherosclerotic disease [24]. To adjust for cholesterol exposure, we divided the atherosclerotic lesion size by TC concentrations (Fig. 6B). As a result, the corrected atherosclerotic lesion size of ABCA1 transplanted mice was 3.1-fold increased compared to WT controls, underscoring the important anti-atherogenic role for macrophage ABCA1 in atherosclerotic lesion development. Furthermore, since TC levels did not differ between apoE KO transplanted mice and WT controls, the corrected lesion size also showed a similar anti-atherogenic role for macrophage apoE. Importantly, dKO transplanted mice displayed a 4.3-fold increased lesion development compared to WT controls after TC adjustment.

To examine the relative contribution of both genes to atherosclerotic lesion development, independent of effects on TC levels, two-way ANOVA was used on the corrected atherosclerosis data to analyze possible interactions between macrophage ABCA1 and apoE expression. The regular P value was used to express the significance of the effect of apoE deletion on ABCA1 and vice versa, whereas the interaction P value shows the independent effects of both genes on atherosclerotic lesion development. This test showed highly independent effects of ABCA1 or apoE deletion in bone marrow-derived cells on atherosclerotic lesion formation (Fig. 6B). The apoE gene accounted for 10% of the variation (p = 0.0008), whereas the ABCA1 gene contributed 59% to the total variation (p<0.0001). In agreement, no interaction (p = 0.37) was observed, suggesting that single deletion of ABCA1 or apoE induces atherosclerosis, independent of cholesterol levels. Moreover, atherosclerotic lesion development in dKO transplanted mice is an additive, independent effect of the single deletions of ABCA1 and apoE.

### *In Vitro* Cellular Cholesterol Efflux

**Figure 7:**
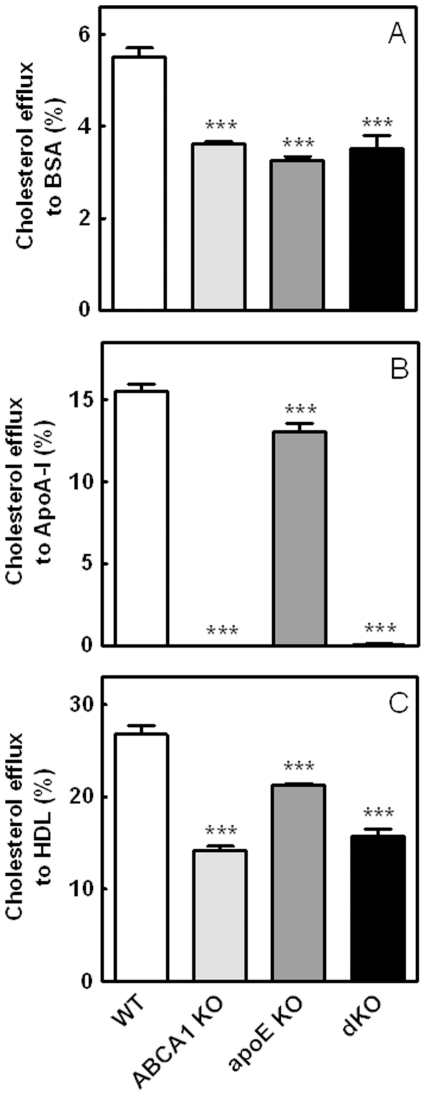
*In vitro* cellular cholesterol efflux. Cholesterol efflux from bone marrow-derived macrophages (BMDM) to BSA (A), apoA-I (10 µg/mL; B) and HDL (50 µg/mL; C) was determined after 24 h. Data are expressed as the percentage of radioactivity released into the medium. Values represent the means of n≥3 mice ± SEM per group. ***p<0.001 as compared to WT transplanted controls.

Combined deletion of macrophage ABCA1 and apoE resulted in a significant increase in atherosclerotic lesion development. Moreover, spleens of these mice were significantly enlarged and enriched in CE content. To determine whether these effects were caused by a defect in the ability to transport cholesterol out of the cells to exogenous lipid acceptors, an *in vitro* cholesterol efflux assay to BSA, apoA-I, and HDL was performed. In line with previous results [5], deletion of macrophage apoE resulted in an impaired cholesterol efflux to BSA (41%, p<0.001; Fig. 7A) emphasizing its ability to promote the efflux of cellular cholesterol in the absence of extracellular acceptors. Similarly, a 35% decreased cholesterol efflux to BSA was observed with ABCA1 KO and dKO cells (p<0.001). As expected, cholesterol efflux to apoA-I was completely inhibited when macrophages lacking ABCA1 were used (p<0.001; Fig. 7B). ABCA1 KO macrophages also exhibited a 47% decreased efflux capacity to HDL (p<0.001; Fig. 7C). In agreement with previous data [5], [12], deficiency of apoE in macrophages resulted in a small but significant decrease in cholesterol efflux to both apoA-I (16%, p<0.001), and HDL (21%; p<0.001). However, surprisingly, no additional effect of apoE deficiency was observed on cholesterol efflux to HDL in the absence of macrophage ABCA1, suggesting that efflux induced by endogenously produced apoE requires ABCA1 expression. It also indicates that the enlarged lipidated spleen and the enhanced atherosclerotic lesion development specifically observed in dKO transplanted mice cannot be explained by additional effects on the cholesterol efflux mechanisms.

### Increased Expression of Hepatic and Systemic Pro-Inflammatory Cytokines

**Figure 8:**
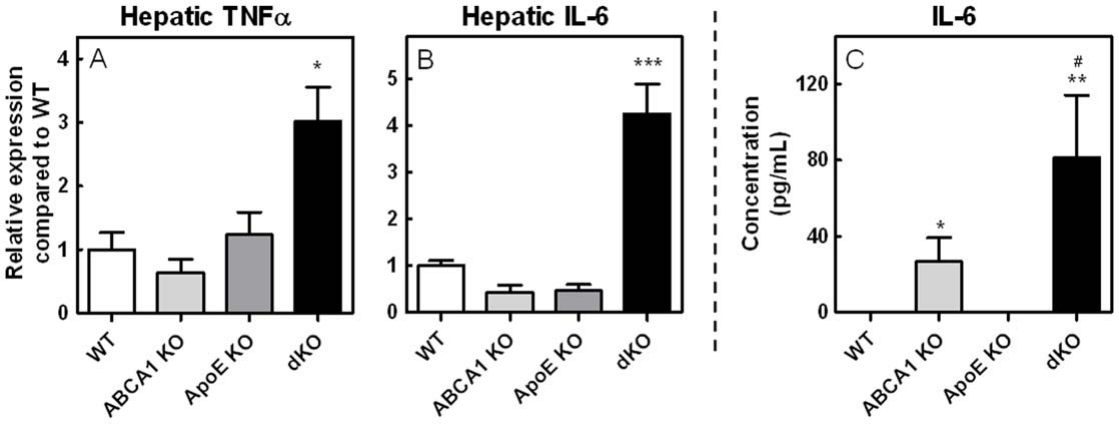
Hepatic gene expression levels and circulating IL-6 levels. mRNA expression of TNFα (A), and IL-6 (B) in liver from bone marrow transplanted LDLr KO mice. Gene expression analysis was performed by RT-PCR. Values are relative to the average expression of housekeeping genes and expressed as fold induction compared to WT transplanted controls. Values are mean ± SEM, n≥3 mice per group as compared to WT. Increased circulating IL-6 levels in LDLr KO mice transplanted with ABCA1/apoE double KO bone marrow (C). Serum from the transplanted LDLr KO mice was collected at 17 weeks after transplantation including 9 weeks of WTD feeding. Values are mean ± SEM, n≥8 mice per group. *p<0.05, **p<0.01. ***p<0.001 as compared to WT transplanted controls. ^#^p<0.05 as compared to ABCA1 KO transplanted animals.

In order to further investigate the possible mechanisms regarding the increased atherosclerotic lesion development in ABCA1/apoE dKO transplanted mice, we quantified mRNA expression levels of the inflammatory molecules TNFα and IL-6 in the liver. Surprisingly, we found highly increased TNFα (3.0-fold; p<0.05; Figure 8A) and IL-6 (4.3-fold; p<0.001; Figure 8B) expression levels, indicating an enhanced inflammatory status of these animals. In addition, we analyzed both pro- and anti-inflammatory cytokines in the circulation, including TNFα, IFNγ, IL-6 and IL-10. Whereas TNFα, IFNγ, and IL-10 concentrations were too low to be detected in the serum of these animals, a major increase in the secretion of the pro-inflammatory cytokine IL-6 in ABCA1 KO transplanted animals was observed (p<0.05; Figure 8C), which was even further enhanced in dKO transplanted animals (3.1-fold as compared to ABCA1 KO transplanted animals; p<0.05). Serum IL-6 is generally considered a systemic inflammatory marker. As a result, the observed elevated IL-6 levels indicate an intensified inflammatory environment which, at least partly, contributed to the observed increase in atherosclerotic lesion development.

## Discussion

Although the functions of macrophage ABCA1 and apoE in the development of atherosclerosis have been studied extensively [3], [4], [5], [12], [13], [14], [15], their potential combined role in atherosclerotic lesion development is still unexplored. In the present study, we show that macrophage ABCA1 and apoE independently protect against atherosclerotic lesion formation, when normalized for serum cholesterol exposure.

ABCA1 is known to facilitate cholesterol efflux to lipid-poor apolipoproteins [6]. As a result, cholesterol efflux to apoA-I was completely abolished upon deletion of ABCA1 in macrophages. Macrophages do not produce apoA-I, but, apoE secreted by macrophages [10] is able to induce efflux of cholesterol both in the presence and absence of extracellular cholesterol acceptors (e.g. ApoA-I or HDL) [11], which is confirmed in this study. Interestingly, no added effect on cholesterol efflux was observed in dKO macrophages compared to ABCA1 deficient macrophages, indicating that endogenously produced apoE requires ABCA1 to facilitate cholesterol efflux.

Nonetheless, spleens of ABCA1/apoE dKO transplanted mice were enlarged due to severe CE accumulation. In agreement with the current study, several publications have shown increased lesion development in ABCA1 KO transplanted mice, despite a clear reduction in serum cholesterol levels [3], [23], [25]. Interestingly, TC levels in serum were markedly increased upon combined deletion of macrophage ABCA1 and apoE as compared to single deletion of ABCA1 in macrophages. This thus leads to ABCA1 deficiency – a defect in cholesterol efflux – combined with elevated serum total cholesterol levels. Therefore, the observed enlargement and CE accumulation of spleens from dKO transplanted mice are most likely the result of enhanced lipid uptake due to the increased TC pressure from the circulation. In agreement with this hypothesis, less lipid accumulation and no changes in spleen size were observed in single KO transplanted animals.

In the current study, transplanted LDLr KO mice were fed a HF/HC diet for 9 weeks to induce increased serum cholesterol levels. A minimum serum cholesterol concentration of >300 mg/dL is required for the development of experimental atherosclerosis in LDLr KO mice [26]. Single deletion of macrophage ABCA1 or apoE led to a comparable increase of atherosclerotic lesion development. However, circulating TC levels were over 2-fold higher in apoE KO transplanted animals compared to ABCA1 KO transplanted mice. Accordingly, although the absolute lesion size did not differ between both genotypes, the lesions adjusted for serum total cholesterol exposure showed the high relative importance of ABCA1 deficiency for determining atherosclerosis susceptibility. Strikingly, despite the fact that single macrophage apoE deficiency did not alter serum TC concentrations, dKO transplanted mice had significantly higher TC levels than single macrophage ABCA1 KO transplanted animals.

We previously found that circulating apoE levels can be influenced by macrophage ABCA1 deficiency [3]. In addition, ABCA1 was shown to promote macrophage apoE secretion in primary human monocyte-derived macrophages and THP-1 cells [9]. However, in the present study, the amount of apoE on VLDL and LDL particles was not reduced upon transplantation of apoE or ABCA1 deficient bone marrow into LDLr KO mice, indicating no direct quantitative effects of macrophage ABCA1 deficiency on apoE availability.

To get insight into the mechanisms behind the altered TC levels, key genes involved in lipoprotein metabolism were analysed in liver. Of all genes analysed only LPL was found to be differentially expressed in liver. Therefore, it is tempting to speculate that the lower serum TC levels in ABCA1 KO transplanted animals might be the result of the observed increase in hepatic LPL expression levels. LPL is essential for the lipolysis of lipoproteins. Induction of LPL leads to rapid clearance of VLDL and chylomicrons [27]. Therefore, the observed attenuation of LPL expression in the dKO transplanted mice as compared to ABCA1 KO transplanted animals might have contributed to the increased TC levels.

Besides the general significance of serum TC levels, the distribution over the different lipoproteins in the circulation can vary greatly, thereby changing its impact on atherosclerotic lesion development. Non-HDL is considered to have a pro-atherogenic function, whereas HDL – involved in RCT – acts as an anti-atherogenic lipoprotein [2], [28], [29]. Serum lipoprotein distribution analysis revealed that dKO transplanted mice not only showed an increase in the pro-atherogenic non-HDL cholesterol levels, but also exhibited a decrease in anti-atherogenic HDL cholesterol which will probably also have contributed to the enhanced lesion development in the dKO transplanted animals.

Two-way ANOVA analysis showed that the effects of ABCA1 and apoE on lesion development, when normalized for serum TC levels, were independent of each other. This is surprising since apoE can induce cholesterol efflux via ABCA1 and our *in vitro* studies using the single and dKO macrophages clearly showed that ABCA1 is required to induce efflux via endogenously produced apoE.

Importantly, ABCA1/apoE dKO transplanted mice exhibited a large induction of the pro-inflammatory cytokines IL-6 and TNFα in the liver. In addition, serum IL-6 levels were also highly increased in these animals, indicating an enhanced inflammatory status of these animals. The pro-inflammatory cytokine IL-6 is reported to promote endothelial dysfunction, smooth muscle cell proliferation and migration as well as recruitment and activation of inflammatory cells [30]. Moreover, it induces the formation of macrophage-derived foam cells; a key feature of early and advanced atherosclerotic lesion formation [31].

In conclusion, LDLr KO mice transplanted with either ABCA1 or apoE KO bone marrow exhibited a distinct increase in atherosclerosis compared to WT transplanted animals. Importantly, combined deletion of macrophage ABCA1 and apoE results in a defect in cholesterol efflux and, compared to ABCA1 KO transplanted mice, elevated serum total cholesterol levels, and enhanced systemic and hepatic inflammation, together resulting in the observed augmented atherosclerotic lesion development.

## References

[1] Jessup W, Gelissen IC, Gaus K, Kritharides L (2006). Roles of ATP binding cassette transporters A1 and G1, scavenger receptor BI and membrane lipid domains in cholesterol export from macrophages.. Curr Opin Lipidol.

[2] von Eckardstein A, Nofer JR, Assmann G (2001). High density lipoproteins and arteriosclerosis. Role of cholesterol efflux and reverse cholesterol transport.. Arterioscler Thromb Vasc Biol.

[3] van Eck M, Bos IS, Kaminski WE, Orso E, Rothe G (2002). Leukocyte ABCA1 controls susceptibility to atherosclerosis and macrophage recruitment into tissues.. Proc Natl Acad Sci U S A.

[4] Van Eck M, Singaraja RR, Ye D, Hildebrand RB, James ER (2006). Macrophage ATP-binding cassette transporter A1 overexpression inhibits atherosclerotic lesion progression in low-density lipoprotein receptor knockout mice.. Arterioscler Thromb Vasc Biol.

[5] Lammers B, Out R, Hildebrand RB, Quinn CM, Williamson D (2009). Independent protective roles for macrophage Abcg1 and Apoe in the atherosclerotic lesion development.. Atherosclerosis.

[6] Oram JF, Lawn RM, Garvin MR, Wade DP (2000). ABCA1 is the cAMP-inducible apolipoprotein receptor that mediates cholesterol secretion from macrophages.. J Biol Chem.

[7] Kennedy MA, Barrera GC, Nakamura K, Baldan A, Tarr P (2005). ABCG1 has a critical role in mediating cholesterol efflux to HDL and preventing cellular lipid accumulation.. Cell Metab.

[8] Wang N, Lan D, Chen W, Matsuura F, Tall AR (2004). ATP-binding cassette transporters G1 and G4 mediate cellular cholesterol efflux to high-density lipoproteins.. Proc Natl Acad Sci U S A.

[9] Von Eckardstein A, Langer C, Engel T, Schaukal I, Cignarella A (2001). ATP binding cassette transporter ABCA1 modulates the secretion of apolipoprotein E from human monocyte-derived macrophages.. FASEB J.

[10] Mahley RW (1988). Apolipoprotein E: cholesterol transport protein with expanding role in cell biology.. Science.

[11] Lin CY, Huang ZH, Mazzone T (2001). Interaction with proteoglycans enhances the sterol efflux produced by endogenous expression of macrophage apoE.. J Lipid Res.

[12] Van Eck M, Herijgers N, Vidgeon-Hart M, Pearce NJ, Hoogerbrugge PM (2000). Accelerated atherosclerosis in C57Bl/6 mice transplanted with ApoE-deficient bone marrow.. Atherosclerosis.

[13] Fazio S, Babaev VR, Murray AB, Hasty AH, Carter KJ (1997). Increased atherosclerosis in mice reconstituted with apolipoprotein E null macrophages.. Proc Natl Acad Sci U S A.

[14] Boisvert WA, Curtiss LK (1999). Elimination of macrophage-specific apolipoprotein E reduces diet-induced atherosclerosis in C57BL/6J male mice.. J Lipid Res.

[15] Shi W, Wang X, Wong J, Hedrick CC, Wong H (2004). Effect of macrophage-derived apolipoprotein E on hyperlipidemia and atherosclerosis of LDLR-deficient mice.. Biochem Biophys Res Commun.

[16] Zanotti I, Pedrelli M, Poti F, Stomeo G, Gomaraschi M Macrophage, but not systemic, apolipoprotein E is necessary for macrophage reverse cholesterol transport in vivo.. Arterioscler Thromb Vasc Biol.

[17] Huang ZH, Lin CY, Oram JF, Mazzone T (2001). Sterol efflux mediated by endogenous macrophage ApoE expression is independent of ABCA1.. Arterioscler Thromb Vasc Biol.

[18] Santamarina-Fojo S, Peterson K, Knapper C, Qiu Y, Freeman L (2000). Complete genomic sequence of the human ABCA1 gene: analysis of the human and mouse ATP-binding cassette A promoter.. Proc Natl Acad Sci U S A.

[19] Out R, Hoekstra M, Hildebrand RB, Kruit JK, Meurs I (2006). Macrophage ABCG1 deletion disrupts lipid homeostasis in alveolar macrophages and moderately influences atherosclerotic lesion development in LDL receptor-deficient mice.. Arterioscler Thromb Vasc Biol.

[20] Bligh EG, Dyer WJ (1959). A rapid method of total lipid extraction and purification.. Can J Med Sci.

[21] Redgrave TG, Roberts DC, West CE (1975). Separation of plasma lipoproteins by density-gradient ultracentrifugation.. Anal Biochem.

[22] Chomczynski P, Sacchi N (1987). Single-step method of RNA isolation by acid guanidinium thiocyanate-phenol-chloroform extraction.. Anal Biochem.

[23] Out R, Hoekstra M, Habets K, Meurs I, de Waard V (2008). Combined deletion of macrophage ABCA1 and ABCG1 leads to massive lipid accumulation in tissue macrophages and distinct atherosclerosis at relatively low plasma cholesterol levels.. Arterioscler Thromb Vasc Biol.

[24] Castelli WP, Anderson K, Wilson PW, Levy D (1992). Lipids and risk of coronary heart disease. The Framingham Study.. Ann Epidemiol.

[25] Aiello RJ, Brees D, Bourassa PA, Royer L, Lindsey S (2002). Increased atherosclerosis in hyperlipidemic mice with inactivation of ABCA1 in macrophages.. Arterioscler Thromb Vasc Biol.

[26] Getz GS, Reardon CA (2006). Diet and murine atherosclerosis.. Arterioscler Thromb Vasc Biol.

[27] Eckel RH (1989). Lipoprotein lipase. A multifunctional enzyme relevant to common metabolic diseases.. N Engl J Med.

[28] Holewijn S, den Heijer M, Swinkels DW, Stalenhoef AF, de Graaf J (2010). Apolipoprotein B, non-HDL cholesterol and LDL cholesterol for identifying individuals at increased cardiovascular risk.. J Intern Med.

[29] Sweetnam PM, Bolton CH, Yarnell JW, Bainton D, Baker IA (1994). Associations of the HDL2 and HDL3 cholesterol subfractions with the development of ischemic heart disease in British men. The Caerphilly and Speedwell Collaborative Heart Disease Studies.. Circulation.

[30] Keidar S, Heinrich R, Kaplan M, Hayek T, Aviram M (2001). Angiotensin II administration to atherosclerotic mice increases macrophage uptake of oxidized ldl: a possible role for interleukin-6.. Arterioscler Thromb Vasc Biol.

[31] Takeda N, Manabe I, Shindo T, Iwata H, Iimuro S (2006). Synthetic retinoid Am80 reduces scavenger receptor expression and atherosclerosis in mice by inhibiting IL-6.. Arterioscler Thromb Vasc Biol.

